# Awareness and Its Associated Factors of Obstetrics Fistula among Antenatal Care Attendees in Injibara Town Health Institutions, Awi Zone, North West, Ethiopia, 2019

**DOI:** 10.1155/2020/7306108

**Published:** 2020-07-01

**Authors:** Wondu Feyisa Balcha, Azezu Asres Nigussie, Fentahun Yenealem Beyene, Azimeraw Arega Tesfu

**Affiliations:** Department of Midwifery, College of Medicine and Health Sciences, Bahir Dar University, Bahir Dar, Ethiopia

## Abstract

**Background:**

Obstetric fistula is abnormal passageway between the vagina and bladder or rectum, and it has the most devastating effects on physical, social, and economic levels and represents a major public health issue of thousands of women, which failed to provide accessible and appropriate intrapartum care for women within a developing country, particularly in Ethiopia. Therefore, we tried to assess the awareness and its associated factors of obstetrics fistula among pregnant mothers attending antenatal care clinics.

**Methods:**

A health institutional-based cross-sectional study was employed from March 4 to 29/2019 among 413 pregnant women. Data was collected by a systematic random sampling technique and entered into a computer using Epi data 3.5, edited and analyzed using Statistical Package of Social Sciences 23.0 version. Bivariate and multivariate logistic regression analyses were employed to estimate the crude and adjusted odds ratio with a confidence interval of 95% and *p* value of less than 0.05 considered statically significant.

**Result:**

This study identified that 39.5% with 95% confidence interval (34.6-44.6%) of pregnant women had good awareness about obstetrics fistula. Multivariate logistic regression analysis showed that living in urban [*AOR* = 1.98, 95% *CI* = 1.07 − 3.69], attending formal education [*AOR* = 2.11, 95% *CI* = 1.06 − 4.12], having history antenatal care [*AOR* = 3.87, 95% *CI* = 1.60 − 9.68], and childbirth at health institution [*AOR* = 7.10, 95% *CI* = 2.52 − 2.02] were significantly associated with awareness of obstetrics fistula. *Conclusion and recommendation.* This study showed that awareness of obstetrics fistula was low. Residency, education, and occupation of the women, having history of antenatal care and childbirth at health institution was significantly associated with awareness of obstetrics fistula. Still, there is a gap on awareness of obstetrics fistula; therefore, it is good to emphasize on providing information on maternal health care issues, particularly about obstetrics fistula.

## 1. Background

Obstetrics fistula is a complication that arises from prolonged or obstructed labor without prompt medical care which causes tissue necrosis resulting in a hole between the vagina and bladder or rectum, or both [1]. It is a public health issue for women and their communities within developing settings, particularly in Africa and Southeast Asia. Of which signifies a health system that has failed to provide accessible, timely, and appropriate intrapartum care [2, 3]. Globally, around two million, women living with untreated fistulas, and yearly, there are between 50,000 to 100,000 new cases of OBF develop worldwide. Of these, around 33,000 women live in Sub-Saharan Africa, and it affects about 1.57 per 1,000 women in Sub-Saharan Africa and 1.62 women per 1000 reproductive age in Ethiopia [4]. In prolonged obstructed labor vaginal, bladder, and rectal damage results from compression of the maternal tissue by the fetus during repeated uterine contractions that restrict blood flow, resulting in ischemia and tissue damage and which results in continuous and uncontrolled leakage of urine [5]. OBF primarily affects lower socioeconomic classes, who are under privileged, underemployed, and have limited access to safe delivery attended by qualified health personnel [6]. The underlying factors contributing to obstetrics fistula (OBF) include no skilled birth attendants, poor health-seeking behavior, poor referral systems and transportation network, inadequate facilities providing comprehensive obstetric care services, poverty, malnutrition, lack of education, early marriage and childbirth, harmful traditional practices, sexual violence, and lack of good quality or accessible maternal and health care [7–10]. To 2016, Ethiopian Demographic Health Survey (EDHS), the national prevalence of obstetric fistula is less than 1% among ever-married women. The highest prevalence (0.7%) occurs in the Amhara region [11]. OBF is treatable by surgery, by giving palliative care and can be prevented completely through timely access to competent emergency obstetric care as providing support from trained health care professionals throughout pregnancy, use of pantograph in all labor, providing access to family planning, promoting birth spacing, and postponing early marriage and by educating the communities and promoting education for girls are the key factors to prevent in long term [2–4, 12]. Therefore, we tried to assess awareness of OBF and its associated factors.

## 2. Methods

### 2.1. Study Area

The study was carried out in Injibara town which is found in the Southwestern part of the Amhara region and north western part of Ethiopia. The town is located about 447 km away from the capital city of Ethiopia which is Addis Ababa and 118 km away from Amhara regional state Bahir Dar city. Injibara is the administrative town of Awi zone. The town has five Kebeles with the population of 46,745 (23,466 females and 23,279 are males). The town has 2 governmental health facilities [13].

#### 2.1.1. Study Design and Period

A health facility-based cross-sectional study was conducted from March 4 to 29/2019 in Injibara town, Awi zone, Ethiopia.

#### 2.1.2. Source of Population

All pregnant women who came to attend ANC at all government health facilities in Injibara town.

#### 2.1.3. Study Population

Pregnant women who attended ANC at selected governmental health facilities in Injibara town.

#### 2.1.4. Inclusive Criteria

Pregnant women who were attended ANC in the governmental health facility at Injibara town.

#### 2.1.5. Exclusive Criteria

Pregnant women who had seriously ill.

#### 2.1.6. Operational Definition

Good awareness: those respondents who scored greater or equal to the mean value questions related to OBF.

Poor awareness: those respondents who scored less than the mean value of the question related to OBF [3].

Obstetrics fistula: obstetrics fistula is an opening in the wall of the vagina connecting to the bladder or rectum due to prolonged or obstructed labor [14].

### 2.2. Sample Size Determination

The sample size was determined using a single population proportion formula by considering the following assumptions: the proportion of awareness on VVF among pregnant women was 57.8% [15], 5% level of significance (*α* = 0.05) and 5% margin of error (*ω* = 0.05). The final sample size was adjusted by adding 10% nonresponse rate thus turned out to be 413.

### 2.3. Sampling Procedure and Technique

All governmental health facilities were considering. Information about the client flow to each health facility was obtained from Awi zonal health bureau [16]. The average client flow of the health facilities was taken from a registry book of the health facilities. The number of pregnant women who have attended ANC quarterly in Injibara general hospital was 1320 and in Injibara health center was 2280. The total sample size was proportionally allocated for each governmental health facility of the town based on their quarterly ANC flow. For each health institution, the first participant selected randomly; then, the subsequent participants were selected by a systematic sampling technique every two interval for each health facility.

### 2.4. Data Collection Tools and Procedures

A structured interviewer-administered questionnaire was used to collect the data which was adapted from relevant literatures and modified to local context [3, 15]. Questionnaires were first prepared in the English language; then, it was translated first into Amharic and then to Awigni by an individual who has good ability of these languages then retranslated back to first to Amharic and then too English to check the consistency. The questionnaire was consisting of socio-demographic characteristics, reproductive and obstetrics characteristics, and knowledge and prevention method's related questionnaires (additional file 1). Pretested, structured interviewer-administered questionnaire was used for data collection purposes. The data was collected by two diploma midwives and supervised by two BSc midwives by those who can speak and write the local language.

### 2.5. Data Quality Assurance

The collected data were checked for the completeness, accuracy, clarity, and consistency after conducting pretest. A pretest was conducted on 5% or 21 pregnant mothers in one of the health centers out of the study area called Lideta health center, and the instrument was amended accordingly. Any error, ambiguity, or incompleteness identified was corrected immediately. The data collectors were trained for one day about the contents of the questionnaire, methods of data collection, and aim of the study. The data collection process was supervised by the supervisor and the investigator throughout the data collection period.

## 3. Data Processing, Analysis, and Interpretation

Data was coded, cleaned, and entered by Epi. Data version 3.5 and analyzed using computer database software and transported to the SPSS version 23 statistical software. Descriptive statistics like frequencies and percentages were used to present the categorical independent variables, and mean/standard deviation was used to describe a continuous variable. Frequency tables and graphs were used to present descriptive results. For this study, a bivariate logistic regression model was fitted as the most method of analysis. Odds ratios (OR) were computed with the 95% confidence interval (CI) to see the awareness of OBF for the considered associated factors in this research. Independent factors, with a *P* value <0.2 obtained in the bivariate logistic regression were entered into the multivariate logistic regression models. Consequently, the most important associated factors were identified using the multivariate logistic regression analysis. Then, an adjusted odds ratio (AOR) with 95% confidence interval was calculated for the most predictive variables, and statistical significance was accepted at (*P* < 0.05).

## 4. Result

### 4.1. Socio-Demographic Characteristics of the Respondents

A total of 413 women participated in the study with a response rate of 100%. The mean age of the study participants was 26.60 years with (±*SD* = 5.072) and ranging from 15 to 40 years. About 41.2% were found in the age group 20-25 years. Around 67.6% of participants were Agew ethnicity, and 83.7% were orthodox Christianity religion followers. Of the study participants, 60% were housewives. From the participants, 30.2% attended primary school, and 34.6% husbands of the respondents had diploma and above educational levels (Table 1).

### 4.2. Obstetrics Characteristics of the Study Participants

From the total participants, 81.5% were married at the age of greater than eighteen, and 89.6% of them were got pregnant first at the age of greater than eighteen with the median age of 20 years. With parity of the total participants, 39.0% of the participants' were nulliparous. Among multipara, 66.8% had no history of abortion, and 63.4% had a history of ANC follow-up in their previous pregnancy. Among those who had a history of birth, 65.9% were given their most recent childbirth at a health facility (Table 2).

### 4.3. Current Awareness of Obstetrics Fistula

In this study, 163 (39.5%) of the participants had good awareness about OBF. The risk factors, symptoms of OBF, and availability of OBF treatment and prevention methods identified by 175 (42.4%), 135 (32.7%), 92 (22.3%), and 148 (35.8%) of the participants, respectively (Table 3).

### 4.4. Determinants of Awareness of Obstetrics Fistula

Bivariate analysis was done to assess any relation between independent variables and awareness of obstetrics fistula. In bivariate analysis, age, residency, education status of the respondents, age at first marriage, age at first pregnancy, parity, history of ANC, place of delivery, utilization of PNC, and utilization of modern FP were considered statistically significant with awareness of obstetrics fistula. Multivariable logistic regression analysis showed that participants who live in urban were 1.98 times more likely aware of OBF than who lives in rural (*AOR* = 1.98, 95% *CI* = 1.07 − 3.69). Likewise, respondents who attend formal education were 2.11 times more likely aware of OBF than those who did not attend (*AOR* = 2.11, 95% *CI* = 1.406 − 4.12). Respondents who had history of ANC follow-up were 3.87 times more likely aware of OBF than those who had no history of ANC follow-up (*AOR* = 3.87, 95% *CI* = 1.60 − 9.38), and those who gave birth in health institution were 7.10 times more likely aware of OBF than who gave birth at home (*AOR* = 7.10, 95% *CI* = 2.52 − 20.02) (Table 4).

## 5. Discussion

In this study, less than half (39.5%) with 95% of (*CI* = 34.6 − 44.6%) of the participants had good awareness about OBF. This result was lower than studies in Northern Ghana, Nigeria, and Tanzania (45.8%, 57.8%, and 60.1%) [17, 18, 15], respectively. The difference might be the difference in the study population, study design, and the difference in socio-demographic characteristics. The awareness levels in this study is in line with a study in Burkina Faso (36.4%) [3]. The closeness of this result might be due to as both studies done in developing country and may have related level of awareness. The figure in this study is in line with 2016; EDHS (39%) were reported as they had heard about OBF at national levels [12]. This closeness of the result might be due to the same socio-demographic characteristic of the respondents and may be due to closeness of the study period. In this study, socio-demographic and obstetric factors were related to awareness of OBF. The odds of having awareness about OBF was higher among respondents who are living in urban were approximately two times higher than who are lives in rural (*AOR* = 1.98, 95% *CI* = 1.07 − 3.69). This finding agree with a study done in Burkina Faso where urban residency was more aware of OBF than rural residency [3]. This might be due to urban women with more availability of information access, easily accessibility of facility, and accessibility of education than rural women. The odds of having awareness about OBF was higher among respondents who attend formal education were two times higher than who did not attend formal education (*AOR* = 2.11, 95% *CI* = 1.06 − 4.12]. The finding in this study agree with to studies done in Burkina Faso and Northern Ghana [3, 17]. This might be those attending formal education have greater opportunity to get information, asking and getting health services than those who had not attended formal education. The odds of having awareness about OBF was higher among study participants who had history of ANC follow-up in their previous pregnancy were approximately four times higher than who had not had ANC follow-up in their previous pregnancy (*AOR* = 3.87, 95% *CI* = 1.60 − 9.38). This might be attributed to that utilization of maternal health care services have greater opportunity to get exposed to health education and to dissemination structured and targeted message on the health of women and utilization of ANC services may help them to get information about OBF. In this study, awareness about OBF was higher among study participants who had gave birth at health institution were seven times higher than those who gave birth at home (*AOR* = 7.10, 95% *CI* = 2.52 − 20.02). This might be that women who had history of maternal and health care services utilization would have high chance of getting information from health personnel in the form of health education or counseling about OBF.

## 6. Conclusion and Recommendation

In this study, pregnant women who had good awareness about OBF were lower than a study done in Nigeria. Residency, educational levels of the respondents, having history of ANC, and place of delivery were having positive association with awareness of OBF. While occupational levels of the respondents have negative association with awareness of OBF. As per finding, improving the level of awareness, by promoting women's education, full utilization of both ANC and intrapartum care, and creating different strategies for rural women, gives a better understanding of MNCH services. We also recommend further researcher to come up with additional and detailed findings especially on qualitative aspect.

## Figures and Tables

**Table 1:** Percentage distribution of the study participants by socio-demographic characteristics in governmental health facilities of Injibara town, North West Ethiopia, 2019, (*n* = 413).

Variables	Categories	Frequency	Percentage
Age	15-19	24	5.8
	20-25	170	41.2
	26-30	148	35.8
	31-35	45	10.9
	36-40	26	6.3
Ethnicity	Agew	279	67.6
	Amhara	116	28.1
	Others^∗^	18	4.3
Residency	Urban	295	71.4
	Rural	118	28.6
Religion	Orthodox	346	83.71
	Muslim	56	3.6
	Protestant	11	2.7
Marital status	Married	410	99.3
	Single	3	0.7
Educational level of the women	Not read and write	108	26.2
	Primary school (1-8)	125	30.2
	Secondary school (9-12)	94	22.8
	Diploma and above	86	20.8
Occupational status of the women	Government employee	58	14.0
	Housewife	248	60.0
	Merchants	83	20.1
	Others^∗∗^	24	5.9
Educational levels of the husband (*n* = 410)	Not read and write	54	13.2
	Primary school (1-8)	115	28.1
	Secondary school (9-12)	99	24.1
	Diploma and above	142	34.6
Occupational status of the husband (*n* = 410)	Government employee	99	24.1
	Farmer	87	21.2
	Private organization employee	40	9.8
	Merchants	146	35.6
	Car driver	23	5.6
	Others^∗∗∗^	15	3.7

^∗^ (Oromo and Gumze), ^∗∗^ (student, private organization employee and day laborer), ^∗∗∗^ (carpenter and day laborer).

**Table 2:** Percentage distribution of the study participants by reproductive and obstetrics characteristics in governmental health facilities of Injibara town, North West Ethiopia, 2019 (*n* = 413).

Variables	Categories	Frequency	Percentage
Age at first marriage (*n* = 410)	<18	73	17.5
	>=18	337	81.5
Age at first pregnancy	<18	43	10.4
	>=18	370	89.6
Gravidity	Primigravida	148	35.8
	Multigravida	265	64.2
Age at first childbirth (*n* = 252)	<18	25	9.92
	>=18	227	90.08
History of abortion (*n* = 265)	Yes	88	33.2
	No	177	66.8
ANC follow-up in previous pregnancy (**n** = 265)	Yes	168	63.4
	No	97	36.6
Place of delivery for recent childbirth (**n** = 252)	Health institution	166	65.9
	Home	86	34.1
Mode of delivery for recent childbirth (**n** = 252)	Spontaneous vaginal delivery	182	72.2
	Instrumental delivery	45	17.1
	Caesarean section delivery	27	10.7
Attendant of the labor (*n* = 252)	Health workers	166	65.9
	TBA	47	18.7
	Family	39	15.4
History of PNC utilization (**n** = 252)	Yes	55	21.8
	No	197	78.2
History of FP utilization	Yes	254	61.5
	No	159	38.5

**Table 3:** Distribution of the study participants according to awareness of obstetrics fistula in governmental health facilities of Injibara town, North West Ethiopia, 2019, (*n* = 413).

Variables	Categories	Frequency	Percentage
Awareness	Good awareness	163	39.5
	Poor awareness	250	60.5
Source of information	Media	65	32.5
	Health professional	80	40
	From school	32	16
	Others^∗^	23	11.5
Awareness of OBF risk factors	Yes	175	42.4
	No	238	57.6
Which risk factors of obstetrics fistula do you know?	Female gentile mutilation	121	29.3
	Early marriage	144	34.9
	Early pregnancy	139	33.6
	Un spaced childbirth	86	20.8
	Prolonged labor	137	33.2
	Home delivery	153	37.1
	Malnutrition of the mothers	63	15.3
	Not using FP methods	99	24.0
Awareness of symptoms of obstetrics fistula	Yes	135	32.7
	No	278	67.3
Symptoms of obstetrics fistula	Urinary incontinency	134	32.5
	Faecal incontinency	70	17.0
	Vulvar irritation	47	11.4
Awareness of availability of obstetrics fistula treatment	Yes	92	22.3
	No	321	77.7
Awareness of obstetrics fistula prevention	Yes	148	35.8
	No	265	64.2

^∗^ (family and friends).

**Table 4:** Logistic regression analysis for awareness of obstetrics fistula among study participants in governmental health facilities of Injibara town, North West Ethiopia, 2019.

Variables	Categories	Awareness of OBF		Crude OR (95% CI)	Adjusted OR (95% CI)	**P** value
		Yes	No			
Age	15-19	6	18	1	1	
	20-25	82	88	2.80 (1.06-7.39)	2.75 (0.98-7.68)	0.054
	26-30	62	86	2.17 (0.81-5.76)	2.68 (0.86-8.36)	0.088
	>=31	13	58	0.67 (0.22-2.03)	1.12 (0.29-4.31)	0.870
Residency	Rural	20	98	1	1	
	Urban	143	152	4.61 (2.71-7.85)	1.98 (1.07-3.69)	0.030
Educational level of the women	No-formal education	17	91	1	1	
	Attended formal education	146	159	4.92 (2.80,8.65)	2.11 (1.06, 4.12)	0.034
Age at first marriage	<18	14	59	1	1	
	>=18	148	189	3.30 (1.77-6.14)	1.02 (0.43-2.40)	0.973
Age at first Pregnancy	<18	8	35	1	1	
	>=18	155	215	3.15 (1.42-6.99)	0.93 (0.26-.3.35)	0.914
Have ANC in previous pregnancy	No	9	88	1	1	
	Yes	82	86	9.3 (4.41-19.73)	3.87 (1.60-9.38)	0.003
Place of delivery	Home	5	81	1	1	
	Health institution	82	84	15.8 (6.10-41.02)	7.10 (2.52-20.02)	<0.001
Previous postnatal care	No	54	143	1		
	Yes	33	22	3.97 (2.13-7.41)	1.34 (0.61-2.91)	0.467
FP utilization	No		116	1	1	
	Yes		134	2.42 (1.56-3.71)	1.13 (0.65-1.97)	0.677

## Data Availability

The data used to support the findings of this study are available from the corresponding author upon request.

## Abbreviations

ANC: Antenatal care
EDHS: Ethiopian Demography Health Survey
FP: Family planning
OBF: Obstetrics fistula
VVF: Vesico vaginal fistula.

## References

[1] de Bernis L. (2007). Obstetric fistula: guiding principles for clinical management and programme development, a new WHO guideline. *International Journal of Gynecology & Obstetrics*.

[2] Tunçalp Ö., Tripathi V., Landry E., Stanton C. K., Ahmed S. (2015). Measuring the incidence and prevalence of obstetric fistula: approaches, needs and recommendations. *Bulletin of the World Health Organization*.

[3] Banke-Thomas A. O., Kouraogo S. F., Siribie A., Taddese H. B., Mueller J. E. (2013). Knowledge of obstetric fistula prevention amongst young women in urban and rural Burkina Faso: a cross-sectional study. *PLoS One*.

[4] World Health Organization https://www.who.int/reproductivehealth/topics/maternal_perinatal/fistula/en/.

[5] Raassen T. J. I. P., Verdaasdonk E. G. G., Vierhout M. E. (2007). Prospective results after first-time surgery for obstetric fistulas in East African women. *International Urogynecology Journal*.

[6] Wall L. L. (2012). Obstetric fistula is a “neglected tropical disease”. *PLoS Neglected Tropical Diseases*.

[7] Velez A., Ramsey K., Tell K. (2007). The Campaign to End Fistula: What have we learned? Findings of facility and community needs assessments. *International Journal of Gynecology & Obstetrics*.

[8] Donnay F., Ramsey K. (2016). Eliminating obstetric fistula: progress in partnerships. *International Journal of Gynecology & Obstetrics*.

[9] Capes T., Ascher-Walsh C., Abdoulaye I., Brodman M. (2011). Obstetric fistula in low and middle income countries. *Mount Sinai Journal of Medicine: A Journal of Translational and Personalized Medicine*.

[10] Miller S., Lester F., Webster M., Cowan B. (2005). Obstetric fistula: a preventable tragedy. *Journal of Midwifery & Women's Health*.

[11] CSACE I., Demographic E., Survey H. (2016). *Addis Ababa, Ethiopia, and Rockville*.

[12] Polan M. L., Sleemi A., Bedane M. M., Lozo S., Morgan M. A. Obstetric fistula. *Essential Surgery: Disease Control Priorities, Third Edition (Volume 1)*.

[13] Central Statistical Service of Ethiopia (2007). *2007 Population and Housing Census: Administrative Report*.

[14] Adler A. J., Fox S., Campbell O. M. R., Kuper H. (2013). Obstetric fistula in southern Sudan: situational analysis and key informant method to estimate prevalence. *BMC Pregnancy and Childbirth*.

[15] Ezeonu P. O., Ekwedigwe K. C., Isikhuemen M. E. (2017). Awareness of obstetric vesicovaginal fistula among pregnant women in a rural hospital. *Advances in Reproductive Sciences*.

[16] Awi Zone Health Bureau (2015). *Awi Zone Health Bureau Annual plan Achievement Report*.

[17] Saeed M. F., Alhassan A., Opare-Asamoah K., Kuubiere C. (2014). A survey on obstetric fistula awareness in Northern Ghana. *European Journal of Experimental Biology*.

[18] Kazaura M., Kamazima R., Mangi E. (2011). Perceived causes of obstetric fistulae from rural southern Tanzania. *African Health Sciences*.

